# Supplementary material for: A 12-Year Experimental Design to Test the Recovery of Butterfly Biodiversity in an Urban Ecosystem: Lessons from the Parc Urbain des Papillons
Source: Insects. 2023 Sep 24;14(10):780. doi: 10.3390/insects14100780 (PMC10607803; doi:10.3390/insects14100780)
Supplement: Supplementary file 1 [file insects-14-00780-s001.zip › insects-2587013-supplementary.pdf]

insects

MDPI

Supplementary Materials: The following supporting information can be downloaded at: www.mdpi.com/xxx/s1

Table S1. Species selected for the thickets, characteristics and butterflies species targeted by this species

Species	Order	Family	Planted / seeded	Host/nectariferous	Targeted butterfly species
Achillea filipendulina Lam.	*Asterales*	*Asteraceae*	Planted	Nectariferous	
Achillea millefolium L.	*Asterales*	*Asteraceae*	Planted	Host/nectariferous	*Melitaea didyma*
Ajuga reptans L.	*Lamiales*	*Lamiaceae*	Planted	Nectariferous	
Agastache foeniculum (Pursh) Kuntze	*Lamiales*	*Lamiaceae*	Planted	Nectariferous	
Aquilegia vulgaris L.	*Ranunculales*	*Ranunculaceae*	Planted	Nectariferous	
Symphiotrichum laeve (L.) Á. Löve & D. Löve	*Asterales*	*Asteraceae*	Planted	Nectariferous	
Aster nova- angliae (L.) G.L. Nesom	*Asterales*	*Asteraceae*	Planted	Nectariferous	
Anthyllis vulneraria L.	*Asterales*	*Asteraceae*	Planted	Host/Nectariferous	*Cupido minimus, Polyommatus dorylas*
Arabis hirsuta (L.) Scop.	*Brassicales*	*Brassicaceae*	Planted	Host/Nectariferous	*Anthocharis cardamines*
Arbutus unedo L.	*Ericales*	*Ericaceae*	Planted	Host/Nectariferous	*Charaxes jasius*
Ballota pseudodictamnus (L.) Benth.	*Lamiales*	*Lamiaceae*	Planted	Nectariferous	
Biscutella laevigata L.	*Brassicales*	*Brassicaceae*	Planted	Host/Nectariferous	*Anthocharis euphenoides, Pieris napi*
Betonica officinalis L.	*Lamiales*	*Lamiaceae*	Planted	Nectariferous	
Buphthalmum salicifolium L.	*Asterales*	*Asteraceae*	Planted	Nectariferous	
Bupleurum fruticosum L.	*Apiales*	*Apiaceae*	Planted	Nectariferous	
Clinopodium nepeta (L.) Kuntze	*Lamiales*	*Lamiaceae*	Planted	Nectariferous	
Cephalaria leucantha (L.) Schrad. ex Roem. & Schult.	*Dipsacales*	*Caprifoliaceae*	Planted	Host/Nectariferous	*Euphydrias aurinia provincialis*
Centranthus ruber (L.) DC.	*Dipsacales*	*Caprifoliaceae*	Planted	Nectariferous	
Coronilla varia L.	*Fabales*	*Fabaceae*	Planted	Host/Nectariferous	*Lysandra bellargus*
Dianthus barbatus L.	*Caryophyllales*	*Caryophyllaceae*	Planted	Nectariferous	
Dipsacus fullonum L.	*Dipsacales*	*Caprifoliaceae*	Planted	Nectariferous	
Euryops sp.	*Asterales*	*Asteraceae*	Planted	Nectariferous	
Foeniculum vulgare Mill.	*Apiales*	*Apiaceae*	Planted	Host/Nectariferous	*Papilio machaon*
Helichrysum italicum (Roth) G.Don	*Asterales*	*Asteraceae*	Planted	Nectariferous	
Hyssopus officinalis L.	*Lamiales*	*Lamiaceae*	Planted	Nectariferous	

Dittrichia viscosa (L.) Greuter	*Asterales*	*Asteraceae*	Planted	Nectariferous	
Lavandula x intermedia Emeric ex Loisel.	*Lamiales*	*Lamiaceae*	Planted	Nectariferous	
Lavandula angustifolia Mill.	*Lamiales*	*Lamiaceae*	Planted	Nectariferous	
Lavandula stoechas L.	*Lamiales*	*Lamiaceae*	Planted	Nectariferous	
Lantana camara L.	*Lamiales*	*Verbenaceae*	Planted	Nectariferous	
Leucanthemum vulgare Lam.	*Asterales*	*Asteraceae*	Planted	Nectariferous	
Lotus corniculatus L.	*Fabales*	*Fabaceae*	Planted	Host/Nectariferous	*Aricia agestis, Cupido argiades, Erynnis tages, Plebejus argus, Celastrina argiolus, Colias crocea, Erynnis tages, Glaucopsyche alexis, Glaucopsyche melanops, Leptotes pirithous, Polyommatus icarus*
Lychnis coronaria (L.) Desr.	*Caryophyllales*	*Caryophyllaceae*	Planted	Nectariferous	
Medicago lupulina L.	*Fabales*	*Fabaceae*	Planted	Host/Nectariferous	*Cupido argiades, Colias crocea, Glaucopsyche alexis, Polyommatus icarus*
Nepeta x faassenii Bergmans ex Stearn	*Lamiales*	*Lamiaceae*	Planted	Nectariferous	
Oenothera lindheimeri (Engelm. & A. Gray) W.L. Wagner & Hoch	*Myrtales*	*Onagraceae*	Planted	Nectariferous	
Origanum vulgare L.	*Lamiales*	*Lamiaceae*	Planted	Nectariferous	
Rosmarinus officinalis L.	*Lamiales*	*Lamiaceae*	Planted	Host/Nectariferous	*Leptotes pirithous*
Salvia microphylla Kunth	*Lamiales*	*Lamiaceae*	Planted	Nectariferous	
Salvia officinalis L.	*Lamiales*	*Lamiaceae*	Planted	Nectariferous	
Santolina chamaecyparissus L.	*Asterales*	*Asteraceae*	Planted	Nectariferous	
Satureja montana L.	*Lamiales*	*Lamiaceae*	Planted	Nectariferous	
Scabiosa columbaria L.	*Dipsacales*	*Caprifoliaceae*	Planted	Nectariferous	
Hylotelephium spectabile (Boreau) H.Ohba	*Saxifragales*	*Crassulaceae*	Planted	Nectariferous	
Silene latifolia Poir.	*Caryophyllales*	*Caryophyllaceae*	Planted	Nectariferous	
Stachys byzantina K. Koch	*Lamiales*	*Lamiaceae*	Planted	Nectariferous	
Syringa vulgaris L.	*Lamiales*	*Oleaceae*	Planted	Nectariferous	
Thymus x *citriodorus* (Pers.) Schreb.	*Lamiales*	*Lamiaceae*	Planted	Host/Nectariferous	*Pseudophilotes baton*
Thymus hirsutus	*Lamiales*	*Lamiaceae*	Planted	Host/Nectariferous	*Pseudophilotes baton*
Thymus longifolium	*Lamiales*	*Lamiaceae*	Planted	Host/Nectariferous	*Pseudophilotes baton*
Thymus vulgaris L.	*Lamiales*	*Lamiaceae*	Planted	Host/Nectariferous	*Pseudophilotes baton*

Urtica dioica L.	*Rosales*	*Urticaceae*	Planted	Host/Nectariferous	*Aglais io, Aglais urticae, Polygonia c-album, Polygonia c-album, Vanessa atalanta, Vanessa cardui*
Verbena bonariensis L.	*Lamiales*	*Verbenaceae*	Planted	Nectariferous	
Xanthoselinum alsaticum (L.) Schur	*Apiales*	*Apiaceae*	Planted	Nectariferous	
Medicago sativa L.	*Fabales*	*Fabaceae*	Seeded	Nectariferous	
Melilotus officinalis (L.) Lam	*Fabales*	*Fabaceae*	Seeded	Nectariferous	
Phacelia tanacetifolia Benth.	*Boraginales*	*Hydro*	Seeded	Nectariferous	
Sinapis alba L.	*Brassicales*	*Brassicaceae*	Seeded	Nectariferous	
Trifolium repens L.	*Fabales*	*Fabaceae*	Seeded	Nectariferous	

Table S2. Inventoried species and their associated IndVal value and frequency

Species	INDVAL value			Relative species frequency			P value
	BUPbefore	BUPafter	WAS	BUPbefore	BUPafter	WAS	
	[2010-2012]	[2018-2022]	[2018-2022]	[2010-2012]	[2018-2022]	[2018-2022]	
Melanargia occitanica			0.8			0.8	0.03
Euchloe crameri		0.333	0.833		0.2	1	0.005
Pontia daplidice		0.114	0.714		0.4	1	0.01
Pieris napi	0.714	0.114		1	0.4		0.039
Gonepteryx cleopatra		0.356	0.556		0.8	1	0.01
Melitaea didyma		0.356	0.556		0.8	1	0.018
Vanessa cardui		0.625	0.225		1	0.6	0.012
Pyronia bathseba	0.725	0.269	0.652	0.333	0.2	1	0.031
Pyronia cecilia	0.476	0.429	0.429	0.333	1	1	0.034
Thymelicus acteon	0.476	0.429	0.429	0.333	1	1	0.044
Aglais urticae			0.2			0.2	1
Fabriciana niobe			0.2			0.2	1
Boloria dia			0.4			0.4	0.285
Coenonympha dorus			0.2			0.2	1
Cupido argiades		0.2			0.2		1
Hipparchia fidia			0.4			0.4	0.301
Pyrgus armoricanus		0.6			0.6		0.077
Pyrgus malvoides		0.6			0.6		0.064
Satyrium ilicis			0.2			0.2	1
Cacyreus marshalli	0.417	0.15		0.667	0.4		0.359
Muschampia baeticus		0.1	0.1		0.2	0.2	1
Muschampia flocciferus		0.45	0.5		0.6	0.2	0.158
Charaxes jasius		0.1	0.1		0.2	0.2	1
Coenonympha pamphilus		0.5	0.45		0.2	0.6	0.185
Cyaniris semiargus		0.267	0.667		0.4	0.2	0.518
Gonepteryx rhamni		0.667	0.267		0.2	0.4	0.501
Leptotes pirithous		0.533	0.133		0.8	0.4	0.159
Libythea celtis	0.152		0.218	0.333		0.4	0.746
Limenitis reducta		0.2	0.2		0.4	0.4	0.582
Pieris mannii		0.257	0.457		0.6	0.8	0.236
Plebejus argus	0.152		0.218	0.333		0.4	0.737
Satyrium esculi		0.5	0.45		0.2	0.6	0.157
Thymelicus sylvestris		0.5	0.45		0.2	0.6	0.151
Aricia agestis	0.197	0.159	0.441	0.667	0.6	1	0.351
Brintesia circe	0.333	0.333	0.333	1	1	1	1
Carcharodus alceae	0.167	0.375	0.375	0.667	1	1	0.253
Celastrina argiolus	0.984	0.353	0.318	0.333	0.2	0.6	0.759
Colias crocea	0.182	0.259	0.455	0.667	0.8	1	0.415
Iphiclides podalirius	0.333	0.333	0.333	1,000	1	1	1
Lampides boeticus	0.641	0.277	0.369	0.333	0.6	0.8	0.544
Lasiommata megera	0.333	0.333	0.333	1	1	1	1
Lycaena phlaeas	0.528	0.469	0.3	0.333	1	0.8	0.117

Maniola jurtina	0.333	0.333	0.333	1	1	1	1
Melanargia galathea	0.357	0.229	0.357	1	0.8	1	1
Ochlodes sylvanus	0.239	0.193	0.193	0.667	0.6	0.6	1
Papilio machaon	0.182	0.259	0.455	0.667	0.8	1	0.444
Pararge aegeria	0.197	0.441	0.159	0.667	1	0.6	0.339
Pieris brassicae	0.182	0.455	0.259	0.667	1	0.8	0.439
Pieris rapae	0.333	0.333	0.333	1	1	1	1
Polyommatus icarus	0.182	0.455	0.259	0.667	1	0.8	0.425
Vanessa atalanta	0.359	0.126	0.316	0.667	0.4	0.2	0.522

Table S3. Species of butterfly present (box grey) and/or absent (box blank) according to the sites

**Species**	**Family**	**BUP_{before}**	**BUP_{after}**	**WAS**
Aglais urticae L.	Nymphalidae			
Fabriciana niobe L.	Nymphalidae			
Aricia agestis Denis & Schiffermüller	Lycaenidae			
Boloria dia L.	Nymphalidae			
Brintesia circe Fabricius	Nymphalidae			
Cacyreus marshallii Butler	Lycaenidae			
Carcharodus alceae Esper	Hesperiidae			
Celastrina argiolus L.	Lycaenidae			
Charaxes jasius L.	Nymphalidae			
Coenonympha dorus Esper	Nymphalidae			
Coenonympha pamphilus L.	Nymphalidae			
Colias crocea Geoffroy *in* Fourcroy	Pieridae			
Cupido argiades Pallas	Lycaenidae			
Cyaniris semiargus Rottemburg	Lycaenidae			
Euchloe crameri Butler	Pieridae			
Gonepteryx cleopatra L.	Pieridae			
Gonepteryx rhamni L.	Pieridae			
Hipparchia fidia L.	Nymphalidae			
Iphiclides podalirius L.	Papilionidae			
Lampides boeticus L.	Lycaenidae			
Lasiommata megera L.	Nymphalidae			
Leptotes pirithous L.	Lycaenidae			
Libythea celtis Laicharting	Nymphalidae			
Limenitis reducta Staudinger	Nymphalidae			
Lycaena phlaeas L.	Lycaenidae			
Maniola jurtina L.	Nymphalidae			
Melanargia galathea L.	Nymphalidae			
Melanargia occitanica Esper	Nymphalidae			
Melitaea didyma Esper	Nymphalidae			
Muschampia baeticus Rambur	Hesperiidae			
Muschampia flocciferus Zeller	Hesperiidae			
Ochlodes sylvanus Esper	Hesperiidae			
Papilio machaon L.	Papilionidae			
Pararge aegeria L.	Nymphalidae			
Pieris brassicae L.	Pieridae			
Pieris mannii Mayer	Pieridae			
Pieris napi L.	Pieridae			
Pieris rapae L.	Pieridae			
Plebejus argus L.	Lycaenidae			
Polyommatus icarus Rottemburg	Nymphalidae			
Pontia daplidice L.	Pieridae			
Pyrgus armoricanus Oberthür	Hesperiidae			
Pyrgus malvoides Elwe & Edwards	Hesperiidae			
Pyronia bathseba Fabricius	Nymphalidae			
Pyronia Cecilia Vallantin	Nymphalidae			
Satyrium esculi Hübner	Lycaenidae			
Satyrium ilicis Esper	Lycaenidae			
Thymelicus acteon Rottemburg	Hesperiidae			
Thymelicus sylvestris Poda	Hesperiidae			
Vanessa atalanta L.	Nymphalidae			
Vanessa cardui L.	Nymphalidae			
